# Effects of Baduanjin exercise on sleep quality, depressive symptoms, quality of life, and body composition in older adults: a systematic review and meta-analysis of randomized controlled trials

**DOI:** 10.3389/fpubh.2026.1882039

**Published:** 2026-07-30

**Authors:** Le Zhao, Mei-Chan Chong, Wan Ling Lee, Weixia Song, Nan Xu, Yan Lou

**Affiliations:** 1 Faculty of Nursing, Universiti Malaya, Kuala Lumpur, Malaysia; 2 Faculty of Health Sciences, Nursing and Education, MAHSA University, Jenjarom, Selangor, Malaysia; 3 Department of Nursing, School of Medicine, Quzhou College of Technology, Quzhou, Zhejiang, China

**Keywords:** Baduanjin, exercise, meta-analysis, older adults, systematic review

## Abstract

**Background:**

Baduanjin, a traditional mind–body exercise, may provide benefits for both physical and psychological health among older adults. The present study systematically evaluated its effects on sleep quality, depressive symptoms, quality of life, body composition, and handgrip strength.

**Methods:**

A systematic search of seven databases was conducted to identify randomized controlled trials investigating Baduanjin among adults aged 60 years or older published before 3 April 2026. Primary outcomes included sleep quality, depressive symptoms, quality of life, body composition, and handgrip strength. The Cochrane Risk of Bias tool (RoB 2.0) was used to evaluate the quality of the study. Standardized mean differences (*SMDs*) or mean differences (*MDs*) with 95% confidence intervals (*CIs*) were used in random-effects meta-analyses. The I^2^ statistic was used to measure between-study heterogeneity, while Egger’s test and funnel plots were used to evaluate publication bias. R (version 4.4.2) was used for all analyses.

**Results:**

Eighteen randomized controlled trials comprising 1,527 participants were included in the final analysis. Pooled analyses showed that Baduanjin significantly improved sleep quality (*MD* = −2.81, 95% *CI*: −3.38 to −2.24, *p* < 0.001) and reduced depressive symptoms (*SMD* = −0.88, 95% *CI*: −1.31 to −0.45, *p* < 0.001). Significant improvements were also observed in quality of life (*SMD* = 0.73, 95% *CI*: 0.29–1.18, *p* = 0.001), handgrip strength (*MD* = 2.11, 95% *CI*: 1.57–2.64, *p* < 0.001), and body composition, including reductions in body mass index, waist circumference, and hip circumference.

**Conclusion:**

Baduanjin, a traditional mind–body exercise, may improve sleep quality, depressive symptoms, quality of life, body composition, and handgrip strength among older adults. Nevertheless, additional high-quality randomized controlled trials are warranted because of methodological limitations and heterogeneity across studies.

**Systematic review registration:**

https://www.crd.york.ac.uk/PROSPERO/view/CRD420261377240, identifier PROSPERO (CRD420261377240).

## 1 Introduction

Population ageing is accelerating globally, with one in six individuals projected to be aged ≥60 years by 2030, and the global older population expected to increase from 1 billion in 2020 to 2.1 billion by 2050, alongside a threefold rise in those aged ≥80 years to 426 million (1). As population ageing continues to accelerate worldwide, the health status and quality of life (QoL) of older adults have emerged as critical global public health issues (2). Accumulating evidence suggests that this population experiences a range of interrelated challenges, including reduced QoL, sleep disturbances, mental health conditions, and unfavorable body composition (e.g., obesity and sarcopenia), which collectively undermine overall health (3). These multidimensional health problems are associated with declines in functional capacity, reduced social participation, and increased risks of chronic diseases, thereby placing substantial burdens on healthcare systems and society (4, 5). Therefore, there is a pressing need to develop safe, effective, and accessible interventions aimed at improving health outcomes in older populations.

At present, management strategies for these conditions mainly consist of pharmacological approaches and psychological interventions. In older populations, however, medication-based treatments are frequently constrained by side effects, drug–drug interactions, and the widespread occurrence of polypharmacy, all of which may diminish both effectiveness and safety (6). While psychological therapies, including cognitive behavioral therapy, have proven beneficial, their application in routine practice is often hindered by inadequate access, financial burden, and persistent social stigma, which can negatively affect patient adherence and engagement (7). Consequently, there is an increasing need to develop non-pharmacological strategies that are safe, practical, and sustainable for long-term use within community settings. Among available health promotion approaches, physical activity has gained substantial interest owing to its broad spectrum of health benefits and is increasingly recognized as an effective means to enhance overall well-being in older adults (8).

In recent years, traditional Chinese exercises have attracted considerable attention as a prominent form of mind–body intervention. Among these, Baduanjin, also referred to as the “Eight-Section Brocade,” stands out due to its characteristic gentle and slow movements, which are coupled with controlled breathing and mental focus, making it particularly beneficial for older adults (9). Originating from over a thousand years of history in traditional Qigong, Baduanjin integrates physical activity, breathing control, and mental regulation through rhythmic and deliberate motions (10). The practice consists of eight movements that are distinct yet interrelated, each one focusing on the coordination of posture, breathing, and mental concentration (11). Overall, Baduanjin is considered a low-intensity exercise that fosters the integration of body and mind while enhancing physical function (12). Compared to other traditional mind–body exercises like Tai Chi and the Five Animal Frolics, Baduanjin is easier to learn, does not require specialized equipment or specific locations, and is accessible to individuals across various age groups and fitness levels, especially older adults committed to long-term exercise (13).

Despite the growing body of literature on Baduanjin in recent years, current evidence remains limited in terms of both depth and research focus. Baduanjin may have positive benefits on outcomes including sleep quality in older persons, according to earlier research (14). Nevertheless, many published systematic reviews have pooled different types of traditional Chinese exercises, including Tai Chi and Qigong, which makes it challenging to distinguish the specific effects attributable to Baduanjin alone (15). Moreover, the majority of existing studies have concentrated on single outcome indicators (e.g., sleep quality), while comprehensive evaluations of multidimensional health outcomes—such as depressive symptoms, quality of life, and body composition—remain scarce (16). Depressive symptoms, quality of life, body composition, and grip strength serve as important indicators of overall health status among older adults and are strongly linked to frailty, functional decline, fall risk, and the process of healthy aging (3–5). Accordingly, comprehensively synthesizing the available evidence regarding the effects of Baduanjin on these multiple dimensions of health outcomes is essential for clarifying the potential value of this traditional exercise in improving both physical and psychological well-being in older populations.

This systematic review was designed to integrate existing evidence and to evaluate the effects of Baduanjin on sleep quality, depressive symptoms, quality of life, body composition, and handgrip strength among older adults. Furthermore, the present analysis specifically explored the influence of Baduanjin on body composition, a domain that remains insufficiently examined in the current literature. By addressing limitations in prior evidence synthesis and the evaluation of multidimensional health outcomes, this study aims to offer more comprehensive and reliable evidence on the role of Baduanjin as a safe and practical non-pharmacological strategy for enhancing overall health in the ageing population.

## 2 Methods

### 2.1 Protocol and guidance

The Preferred Reporting Items for Systematic Reviews and Meta-Analyses 2020 (PRISMA 2020) standards were followed in conducting this study (17). The study protocol was prospectively registered in the PROSPERO database (registration number: CRD420261377240) to improve methodological rigor and guarantee transparency.

### 2.2 Literature search

To achieve broad coverage, both English- and Chinese-language databases were systematically searched. The English-language sources comprised PubMed, Web of Science, the Cochrane Library, Embase, and CINAHL, while the Chinese-language sources included China National Knowledge Infrastructure (CNKI) and Wanfang Data. All databases were searched from their inception until April 3, 2026. The search strategy incorporated controlled vocabulary (e.g., MeSH terms) alongside free-text keywords, and Boolean operators (“AND” and “OR”) were applied to enhance search sensitivity and overall coverage. To improve the precision of the search strategy, terms associated with Baduanjin (intervention) and older adults (population) were applied to identify relevant studies. In addition, related synonyms were incorporated to broaden the search scope across titles, abstracts, and keywords (18). In order to find any records that might have been missed, the reference lists of relevant systematic reviews and eligible studies were also manually searched. Supplementary Table 1 lists each database’s specific search tactics.

### 2.3 Study eligibility criteria

Eligibility criteria were established based on the PICOS framework (Participants, Interventions, Comparators, Outcomes, and Study design).

#### 2.3.1 Types of studies

Only randomized controlled trials (RCTs) were eligible for inclusion. Full-text original studies, including journal articles and academic theses, were considered.

#### 2.3.2 Types of participants

Studies involving adults aged 60 years or older were included.

#### 2.3.3 Types of interventions

Baduanjin had to be the principal intervention, regardless of delivery format (individual- or group-based), supervision status, or use of supporting materials. No restrictions were imposed on intervention frequency, session length, or overall duration. Comparator groups could include usual care, no treatment, health education, routine physical activity, wait-list control, or sedentary behavior.

#### 2.3.4 Types of outcome measures

Eligible studies were required to report at least one outcome of interest, including sleep quality, quality of life, depressive symptoms, handgrip strength, or body composition–related measures.

### 2.4 Criteria for exclusion

Studies were excluded if any of the following criteria were met: (1) participants had a diagnosed severe psychiatric or neurological disorder (e.g., schizophrenia or bipolar disorder); (2) the intervention involved a multicomponent or combined approach (e.g., Baduanjin integrated with Tai Chi, other Qigong styles, or alternative exercise modalities); (3) outcomes were strictly limited to physiological biomarkers or neuroimaging results lacking matching clinical measures; or (4) the article was a non-randomized study, grey literature, or abstract-only publication where full text and primary data remained unavailable after contacting the primary authors.

### 2.5 Study selection and data extraction

For centralized management, documents that were extracted from the seven databases indicated above were imported into EndNote 20. Two reviewers (LZ and WS) independently reviewed the titles and abstracts to eliminate papers that were not pertinent to the research issue after eliminating duplicates. Subsequently, full texts of potentially eligible articles were acquired and thoroughly evaluated by the same reviewers in accordance with predefined inclusion and exclusion criteria. At the full-text stage, the reasons for exclusion were methodically recorded. A third reviewer (NX) was engaged to obtain a final conclusion if disagreements during the selection process could not be resolved through conversation.

Following the study selection procedure, data from each qualifying study were independently retrieved by two reviewers (LZ and WS) and placed into a standard data extraction form. The collected data included general study information (first author, publication year, and study setting), participant characteristics (sample size, age, and sex), intervention details (frequency, session length, total duration, and implementation setting), comparator types, outcome variables and their measurement instruments, as well as any reported adverse events. For continuous outcomes, essential statistical parameters, such as means and standard deviations, were obtained for subsequent meta-analysis. During the data extraction process, disagreements between the two reviewers were settled through conversation to reach an agreement. A third reviewer (NX) was consulted to make the final decision if a consensus could not be achieved.

### 2.6 Quality assessment

Two reviewers (LZ and WS) independently appraised the methodological quality of the included studies using the Cochrane Risk of Bias tool (version 2.0; RoB 2.0) (19). This instrument assesses potential bias in randomized controlled trials across five key domains, namely the randomization process, deviations from intended interventions, missing outcome data, outcome measurement, and selective reporting of results. Each study was categorized as having a low risk of bias, some concerns, or a high risk of bias based on the judgments made across these dimensions. The overall risk-of-bias judgment was used as a primary indicator of study quality (20). In order to reach a consensus, disagreements among the reviewers were discussed; if a third reviewer was required, the final decision was made by NX.

### 2.7 Statistical analysis

The ‘meta’ and ‘metafor’ packages in R4.4.2 were used for all studies. A statistically significant two-tailed *p* value was one that was less than 0.05. Pooled effect sizes were computed using a random-effects model due to the possible clinical and methodological diversity among the included studies. Standardized mean differences (*SMDs*) with 95% confidence intervals (*CIs*) were used to express effect sizes for continuous outcomes when several assessment scales were used. When identical measurement instruments were used, mean differences (*MDs*) with 95% *CIs* were derived. The I^2^ statistic was used to measure statistical heterogeneity between studies (21). Subgroup analyses and meta-regression were used to look into possible sources of heterogeneity. To evaluate the robustness of the pooled estimates, a leave-one-out sensitivity analysis was performed in which each study was sequentially excluded. Publication bias was investigated both numerically using Egger’s regression test and graphically using funnel plots. Additionally, the trim-and-fill method was used to assess and correct for potential publication bias-related funnel plot asymmetry.

## 3 Results

### 3.1 Search results

**Figure 1:**
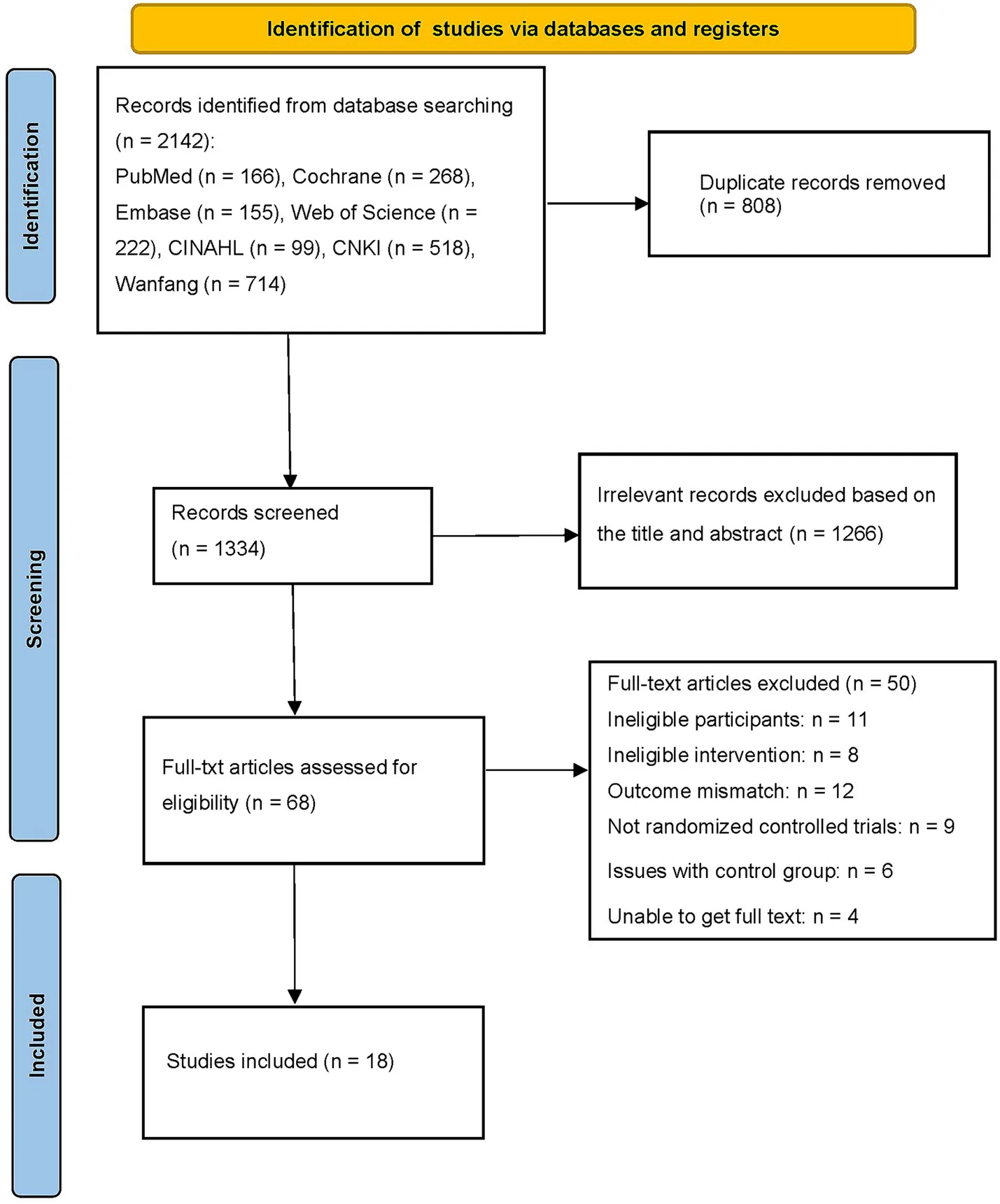
Flow diagram of the study selection process.

According to the predetermined search method, 2,142 records were retrieved from seven electronic databases. Following the removal of 808 duplicate entries, 1,334 records were kept for abstract and title screening. At this stage, 1,266 studies deemed irrelevant to the research objective were excluded. After that, the complete texts of the remaining 68 articles were acquired and their eligibility assessed. During the full-text assessment, 50 articles were excluded. Ultimately, the current systematic review and meta-analysis included 18 studies that satisfied the inclusion criteria (22–39). Figure 1 shows a thorough flow diagram of the study selection procedure.

### 3.2 Characteristics of the included studies

#### 3.2.1 The characteristics of the participants

Of the 18 studies included in this review, the vast majority were carried out in China (*n* = 17) (22–24, 26, 39) (review, the vast majority were carried out in), whereas only one study was conducted in Spain (25). In terms of publication language, four articles were reported in English (23–25, 37), while the remaining studies were published in Chinese (22, 26–36, 38, 39). All eligible studies were designed as RCTs and recruited participants aged 60 years or older. Sample sizes ranged from 32 to 170 participants across the included studies, resulting in a total sample of 1,527 people, of which 764 were in the intervention group and 763 were in the control group. Participants in the intervention groups were between 62.10 ± 0.97 and 78.06 ± 7.14 years old on average, whereas those in the control groups were between 63.50 ± 1.82 and 79.46 ± 5.77 years old. The study populations represented a broad spectrum of health conditions. Specifically, some studies involved generally healthy older adults (23, 25–27, 29, 32, 37), whereas others focused on individuals with sleep disturbances (24, 34, 38) or those diagnosed with chronic conditions, including hypertension, cardiovascular disease, diabetes, and chronic obstructive pulmonary disease (COPD) (22, 28, 30, 31, 33, 35, 36, 39). Further details on the characteristics of the selected studies are given in Table 1.

**Table 1:** Characteristics of the included studies.

Study (author, year)	Country	Sample size	Age(range) or mean ± SD(yr)	Gender (F/M)	Intervention group	Control group	Frequency (min or h/session per/wk)	Duration (weeks)	Training location	Outcome measures	Adverse event
Cao et al. (2016) (22)	China	102 (IG: 52; CG: 50)	IG: 70.83 ± 6.22 CG: 70.14 ± 5.71	40F/62M	Baduanjin qigong	Regular walking	30 min/4 times/w	24 weeks	Community-based Setting	SAS, SDS, FEV₁, FEV₁/FVC, FEV₁%pr	No adverse events reported
Chen et al. (2012) (23)	China	55 (IG: 27; CG: 28)	IG: 70.48 ± 7.90 CG: 72.96 ± 8.30	36F/19M	Baduanjin exercise	Usual activity	30 min/3 times/w	12 weeks	Home	PSQI	No adverse events reported
Fan et al. (2020) (24)	China	139 (IG: 67; CG: 72)	IG: 70.3 ± 5.7 CG: 71.8 ± 6.7	105F/34M	Baduanjin exercise	Usual lifestyle	45 min/5 times/w	24 weeks	Community-based Setting	PSQI, SF-36	No adverse events reported
Fraile et al. (2022) (25)	Spain	117 (EG: 57; CG: 60)	EG: 69.70 ± 6.15 CG: 69.75 ± 6.76	117F/0M	Baduanjin exercise	physical activity	60 min/2 times/w	12 weeks	Community-based Setting	PSQI, HADS	No adverse events reported
Fu et al. (2018) (26)	China	56 (IG: 27; CG: 29)	IG: 68.96 ± 6.89 CG: 69.79 ± 7.30	35F/21M	Baduanjin exercise	Usual Activities	45 min/3 times/w	12 weeks	Community-based Setting	BCAT, Ht, Wt, WC, HC, CC, HGS, BMI, BFP, WHR	No adverse events reported
Guo et al. (2025) (27)	China	103 (IG: 53; CG: 50)	Male: IG: 62.10 ± 0.97 CG: 64.40 ± 1.31 Female: IG: 62.70 ± 1.08 CG: 63.50 ± 1.82	49F/54M	Baduanjin exercise	daily activities	30 min/5 times/w	12 weeks	Community-based Setting	HGS, RT, VC, RHR, BMI, Sit-and-reach, One-leg standing; WHOQOL-BREF	No adverse events reported
Liu et al. (2014) (28)	China	95 (IG: 47; CG: 48)	IG: 67.1 ± 6.18 CG: 66.63 ± 5.98	Not specified	Baduanjin exercise	Walking exercise	30–40 min/7 times/w	12 weeks	Community+Home	SF-36	No adverse events reported
Li et al. (2023) (29)	China	75 (IG: 38; CG: 37)	IG: 71.42 ± 3.94 CG: 73.05 ± 5.40	41F/34M	Baduanjin exercise	Routine community care	40 min/4 time/w	8 weeks	Community-based Setting	B-APQ, GAD-7, PHQ-9, SPPB	No adverse events reported
Shi et al. (2019) (30)	China	57 (IG: 28; CG: 29)	IG: 68.21 ± 4.21 CG: 68.59 ± 4.65	32F/25M	Baduanjin exercise	Walking exercise	30 min/5 time/w	12 weeks	Community-based Setting	FSS, SPPB, BMI, HGS, PSQI, DDS, FBG, 2hPBG	No adverse events reported
Tan et al. (2022) (31)	China	84 (IG: 42; CG: 42)	IG: 68.93 ± 7.21 CG: 68.95 ± 7.21	43F/41M	Baduanjin exercise	Usual Activities	20–30 min/5–7 time/w	12 weeks	Community-based Setting	SBP, DBP, PSQI, antihypertensive response, sleep improvement	No adverse events reported
Tang et al. (2020) (32)	China	32 (IG: 16; CG: 16)	IG: 71.3 ± 5.9 CG: 69.9 ± 4.39	32F/0M	Baduanjin exercise	Monthly health education	60 min/3 time/w	12 weeks	Community activity center	Wt, BFP, muscle mass, bone mass, BMI, energy expenditure, metabolic age, visceral fat; Joint ROM (shoulder, elbow, wrist, neck, hip, knee, ankle)	No adverse events reported
Wang et al. (2016) (33)	China	50 (IG: 25; CG: 25)	60–70 (age range)	24F/26M	Baduanjin exercise	Conventional treatment	hospital + home	12 weeks	Community activity center	Angina (attack frequency, duration); SAS, SDS; SAQ	No adverse events reported
Wang et al. (2019) (34)	China	89 (IG: 45; CG: 44)	IG: 71.50 ± 8.59 CG: 72.31 ± 8.63	49F/40M	Baduanjin exercise	Routine health education	45 min/≥4 times/w	12 weeks	Community-based Setting	PSQI, WMS-RC	No adverse events reported
Wang et al. (2022) (35)	China	93 (IG: 46; CG: 47)	IG: 75.6 ± 8.89 CG: 75.2 ± 8.71	23F/70M	Baduanjin exercise	Conventional treatment	30 min/7 times/w	2 weeks	Hospital ward	PSQI	No adverse events reported
Zheng et al. (2021) (36)	China	68 (IG: 34; CG: 34)	IG: 70.88 ± 5.56 CG: 70.94 ± 6.34	43F/25M	Baduanjin exercise	Conventional treatment	31 min/5 times/w	12 weeks	Community-based Setting	PSQI, salivary cortisol, SBP, DBP, pulse pressure, heart rate	No adverse events reported
Zheng et al. (2018) (37)	China	170 (IG: 85; CG: 85)	IG: 60.53 ± 6.29 CG: 59.75 ± 6.34	109F/61M	Baduanjin exercise	Standard care	60 min/5 times/w	12 weeks	Community centres	Cerebrovascular function (BFV, PI, RI); Cardiopulmonary function (MVV, VC, VVmax, TV, FEV1, IC, FEV1/FVC, echocardiographic parameters); CVD risk factors (TC, TG, LDL-C, HDL-C, FBG, Hcy, BMI, WC, HC, WHR, SBP, DBP, HR); Psychological outcomes (BPOMS, self-confidence, self-esteem, SF-36, PSQI)	No adverse events reported
Ying et al. (2019) (38)	China	74 (IG: 37; CG: 37)	IG: 72.63 ± 3.05 CG: 71.23 ± 4.12	39F/35M	Baduanjin exercise	Routine walking exercise	60 min/5 times/w	12 weeks	Nursing home	PSQI, SAS	No adverse events reported
Guo et al. (2023) (39)	China	68 (IG: 33; CG: 35)	IG: 78.06 ± 7.14 CG: 79.46 ± 5.77	39F/29M	Seated Baduanjin exercise	Conventional limb function exercise and nursing care	30 min/5 times/w	12 weeks	Nursing home	TFI, HGS, MMSE, GDS-30, WHOQOL-BREF, PSQI	No adverse events reported

IG, intervention group; CG, control group; SAS, Self-Rating Anxiety Scale; SDS, Self-Rating Depression Scale; HADS, Hospital Anxiety and Depression Scale; GAD-7, 7-item Generalized Anxiety Disorder Scale; PHQ-9, 9-item Patient Health Questionnaire; B-APQ, Brief Aging Perceptions Questionnaire; BPOMS, Brief Profile of Mood States; SAQ, Seattle Angina Questionnaire; WMS-RC, Wechsler Memory Scale–Revised Chinese version; PSQI, Pittsburgh Sleep Quality Index; SF-36, 36-Item Short Form Health Survey; WHOQOL-BREF, World Health Organization Quality of Life-BREF; BCAT, Basic Cognitive Ability Test; FEV₁, Forced Expiratory Volume in 1 s; FVC, Forced Vital Capacity; FEV₁/FVC, Ratio of Forced Expiratory Volume in 1 s to Forced Vital Capacity; FEV₁%pred, Percent of Predicted Forced Expiratory Volume in 1 s; VC, vital capacity; MVV, maximal voluntary ventilation; TV, tidal volume; IC, inspiratory capacity; SBP, systolic blood pressure; DBP, diastolic blood pressure; HR, heart rate; RHR, resting heart rate; FBG, fasting blood glucose; 2hPBG, 2-h postprandial blood glucose; TC, total cholesterol; TG, triglycerides; LDL-C, low-density lipoprotein cholesterol; HDL-C, high-density lipoprotein cholesterol; Hcy, homocysteine; BMI, body mass index; WC, waist circumference; HC, hip circumference; CC, calf circumference; WHR, waist-hip ratio; BFP, body fat percentage; HGS, handgrip strength; Ht, height; Wt, weight; RT, reaction time; ROM, range of motion; SPPB, Short Physical Performance Battery; FSS, Fatigue Severity Scale; DDS, Diabetes Distress Scale; BFV, blood flow velocity; PI, pulsatility index; RI, resistance index; TFI, Tilburg Frailty Indicator; MMSE, Mini-Mental State Examination; GDS-30, 30-item Geriatric Depression Scale.

#### 3.2.2 The characteristics of the interventions

All eligible studies utilized Baduanjin as the principal intervention. One study implemented a seated version of Baduanjin (39), while the others adopted the conventional standing form (22–38). Intervention protocols differed among studies in terms of frequency and duration. Specifically, each session lasted between 20 and 60 min, with training conducted 2 to 7 times per week over periods ranging from 2 to 24 weeks. Table 1 contains detailed information about the listed research.

#### 3.2.3 Characteristics of controls

Throughout the study period, participants assigned to the control groups in the 18 included studies received either standard care or alternative non-Baduanjin interventions. These interventions encompassed routine care (29, 33, 35–37, 39), walking exercises (22, 28, 30, 38), customary daily activities (23, 24, 26–27, 31), health education (32, 34), and conventional physical activities (25).

#### 3.2.4 Outcome variables

Across the 18 studies included in this analysis, sleep quality emerged as the most commonly reported outcome. To capture the overall effects of Baduanjin, additional outcomes were also investigated, including depressive symptoms, quality of life, and body composition. With respect to sleep-related outcomes, the Pittsburgh Sleep Quality Index (PSQI) was the most frequently applied measurement tool (23–25, 30, 31, 34–39). Psychological outcomes, particularly depressive symptoms, were mainly assessed using validated instruments such as the Self-Rating Depression Scale (SDS), the Hospital Anxiety and Depression Scale (HADS), and the 30-item Geriatric Depression Scale (GDS-30) (22, 25, 33, 39). Measures of quality of life were primarily derived from the 36-Item Short Form Health Survey (SF-36) and the World Health Organization Quality of Life-BREF (WHOQOL-BREF) (24, 27, 28, 39). Furthermore, body composition outcomes were evaluated using indices including body mass index (BMI), waist circumference (WC), and hip circumference (HC) (26, 27, 30, 32, 37). A comprehensive summary of the outcome measures is provided in Table 1.

### 3.3 Risk of bias assessment

**Figure 2:**
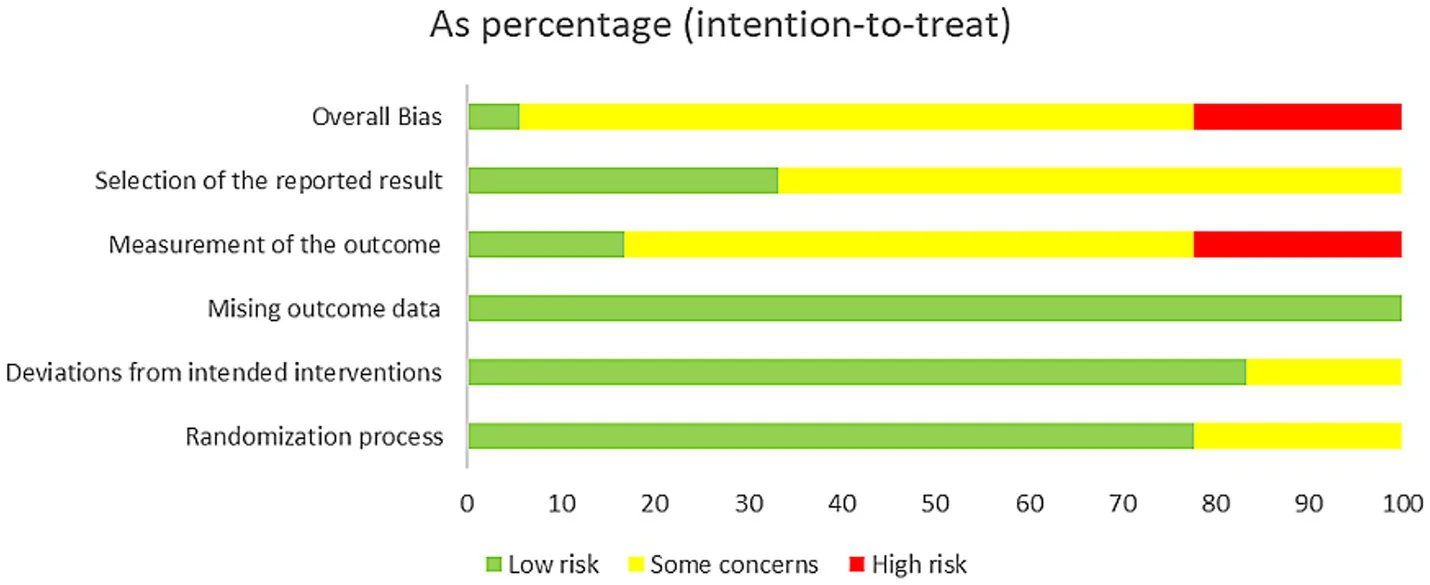
Overall risk of bias: review authors’ judgments about each risk of bias item presented as percentages across all included studies.

**Figure 3:**
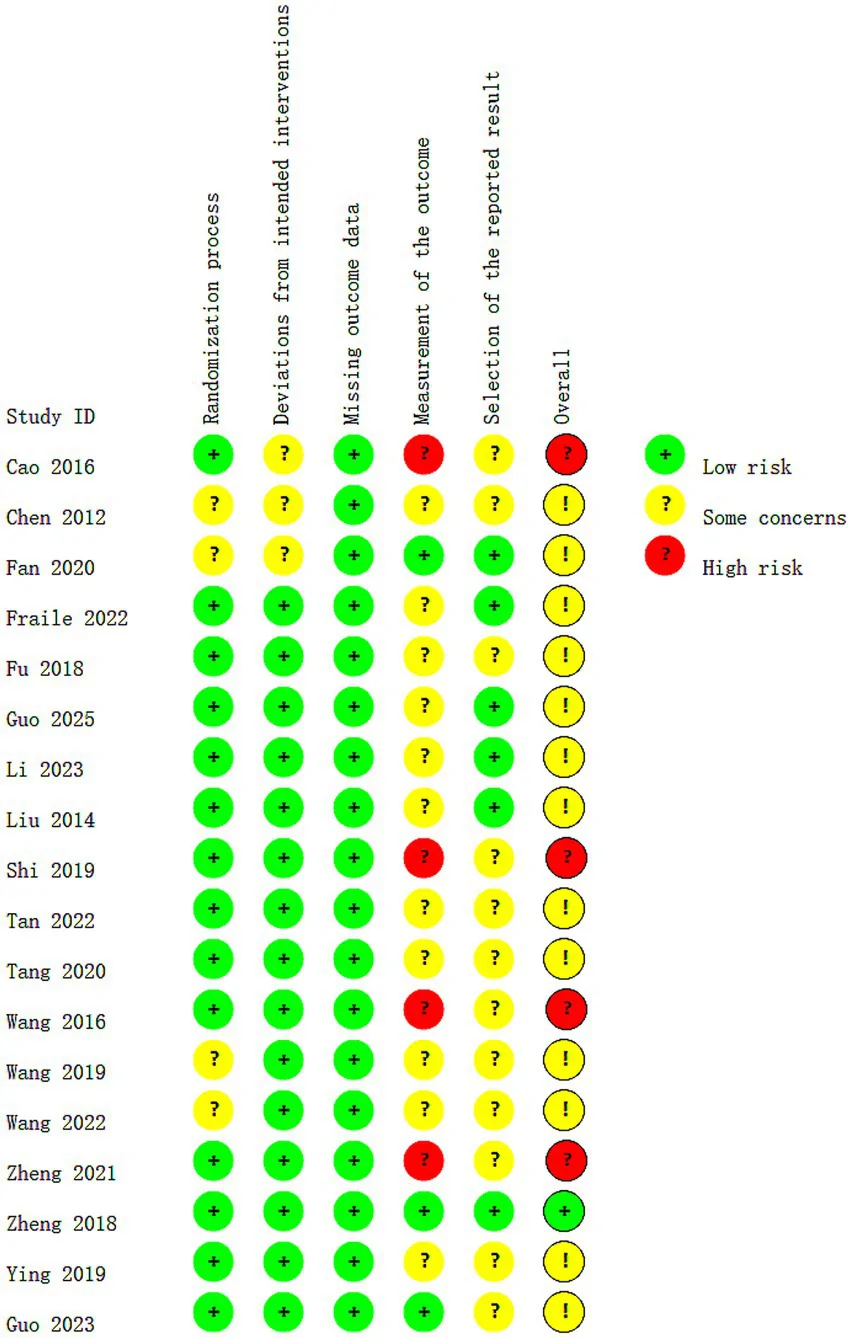
Risk of bias summary.

Across the included studies, the majority adequately described their randomization procedures and were consequently considered to be at low risk of bias in this domain. Nevertheless, a minority of studies did not provide sufficient detail, leading to their classification as having some concerns. For the domain of deviations from intended interventions, most studies were evaluated as low risk, with no clear indications of bias detected. With respect to missing outcome data, all studies were judged to be at low risk of bias, suggesting that follow-up was generally complete and that missing data were unlikely to substantially influence the findings. By contrast, in the domain of outcome measurement, several studies were categorized as having some concerns or a high risk of bias, largely due to inadequate reporting of blinding procedures. In addition, a notable proportion of studies raised some concerns in the domain of selective reporting, which may be attributable to the lack of preregistration or predefined analytical protocols. In summary, although methodological weaknesses were identified in at least one domain for most studies, the overall quality remained acceptable. Specifically, one study was classified as low risk of bias (37), thirteen as having some concerns (23–29, 31, 32, 34, 35, 38, 39), and four as high risk (22, 30, 33, 36). Figures 2, 3 show detailed risk-of-bias assessments for each study.

### 3.4 Results of meta-analysis

#### 3.4.1 Sleep quality

**Figure 4:**
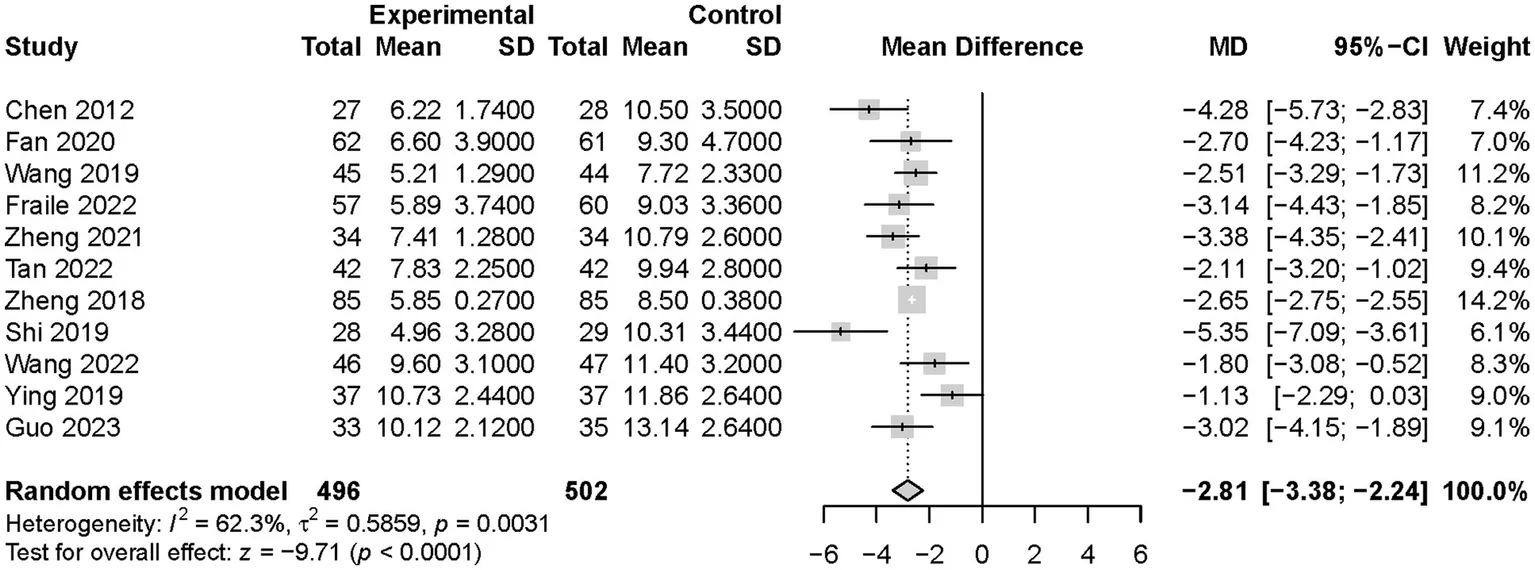
Meta-analysis of Baduanjin exercise on sleep quality among older adults.

A meta-analysis incorporating 11 studies with a total sample size of 998 participants was conducted (23–25, 30, 31, 34–39). The pooled findings indicated that Baduanjin exercise was associated with a significant improvement in sleep quality among older adults, as assessed by the PSQI (*MD* = −2.81, 95% *CI*: −3.38 to −2.24), in comparison with the control group. Furthermore, significant heterogeneity was found in the included studies (I^2^ = 62.3%) (Figure 4).

Supplementary Table 2 summarizes the findings of the subgroup analyses. No statistically significant differences were identified among subgroups defined by health status, session length, or age (*P* for subgroup differences > 0.05). Nevertheless, improvements in sleep quality remained significant within each subgroup, suggesting that the beneficial effects of Baduanjin are generally consistent across varying populations and intervention conditions. To investigate whether intervention duration contributed to the observed heterogeneity across studies, a meta-regression analysis was performed, treating effect size as the dependent variable and intervention duration as the predictor. The analysis revealed no significant relationship between intervention duration and the magnitude of the intervention effect (*β* = 0.020, *p* = 0.423), indicating that intervention duration is unlikely to act as a meaningful moderator. Detailed results are provided in Supplementary Table 3.

Sensitivity analyses confirmed the stability of the pooled estimate, as the overall effect size was not materially altered by the exclusion of any single study (Supplementary Figure 1). Potential publication bias was evaluated using both funnel plot inspection and Egger’s regression test. Visual examination suggested an approximately symmetrical distribution, and no significant small-study effects were found by Egger’s test (t = −0.58, df = 9, *p* = 0.579). Although a minor asymmetry was apparent, subsequent trim-and-fill analysis yielded a more balanced funnel plot without materially changing the pooled estimate. Collectively, these findings suggest that the pooled results were relatively stable; however, the possibility of publication bias should still be interpreted cautiously given the limited number of studies (Supplementary Figure 2).

Data on the individual components of the PSQI were available from four studies. In general, Baduanjin exercise was linked to significant improvements in multiple aspects of sleep when compared with the control condition. Specifically, the intervention led to better subjective sleep quality (*MD* = −0.60, 95% *CI*: −0.74 to −0.46), shorter sleep latency (*MD* = −0.60, 95% *CI*: −0.75 to −0.44), longer sleep duration (*MD* = −0.53, 95% *CI*: −0.64 to −0.41), and higher sleep efficiency (*MD* = −0.71, 95% *CI*: −0.90 to −0.51), with all comparisons reaching statistical significance (*p* < 0.001). Moreover, Baduanjin was linked to reductions in sleep disturbances (*MD* = −0.27, 95% *CI*: −0.39 to −0.16) and decreased reliance on sleep medication (*MD* = −0.24, 95% *CI*: −0.46 to −0.02). Improvements were also observed in daytime dysfunction (*MD* = −0.43, 95% *CI*: −0.66 to −0.21) (Supplementary Figure 3).

#### 3.4.2 Depressive symptoms

**Figure 5:**
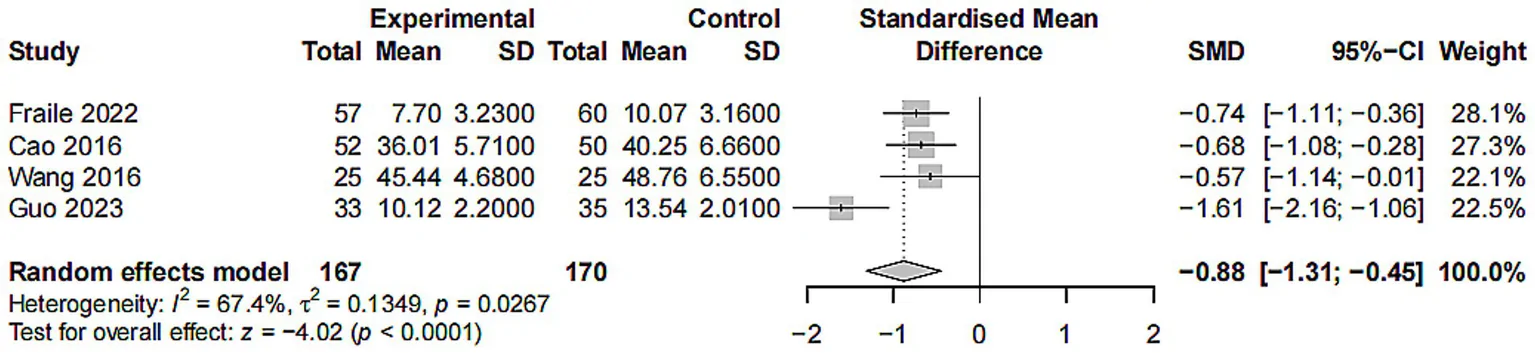
Meta-analysis of Baduanjin exercise on depressive symptoms among older adults.

Four studies comprising 337 participants were incorporated into this meta-analysis (22, 25, 33, 39). Overall, Baduanjin exercise was associated with a significant reduction in depressive symptoms among older adults when compared with the control condition (*SMD* = −0.88, 95% *CI*: −1.31 to −0.45) (Figure 5). Details of the subgroup analyses are provided in Supplementary Table 4. Studies that applied the GDS scale demonstrated a greater effect size than those using the SDS or HADS scales (*SMD* = −1.61, 95% *CI*: −2.16 to −1.06) (Supplementary Figure 4). Moreover, the reduction in depressive symptoms was more pronounced in participants aged ≥70 years than in those aged <70 years (*SMD* = −1.61, 95% *CI*: −2.16 to −1.06) (Supplementary Figure 5). No statistically significant differences were identified across other categorical variables, such as health status (*P* for subgroup differences > 0.05). Sensitivity analysis supported the robustness of the overall results by confirming the stability of the pooled values (Supplementary Figure 6).

#### 3.4.3 Quality of life

**Figure 6:**
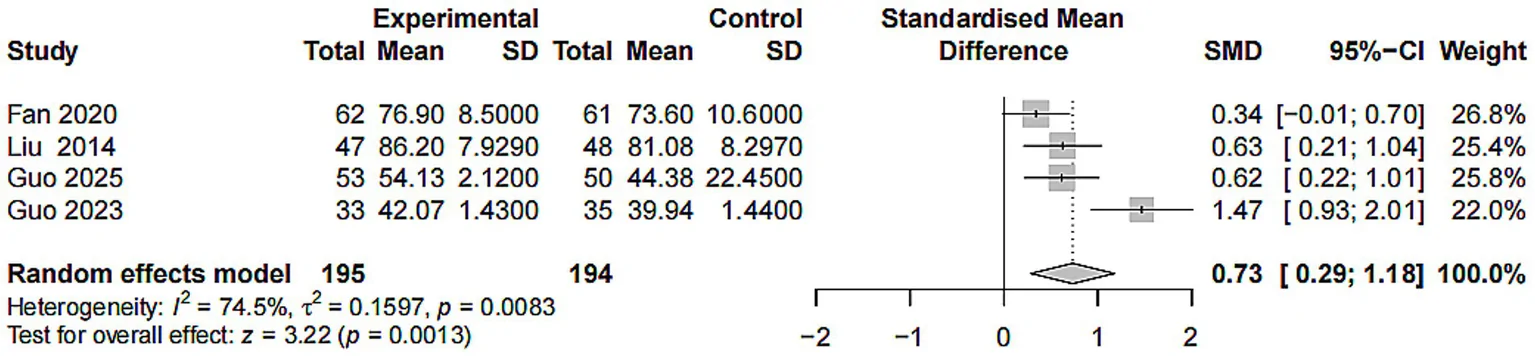
Meta-analysis of Baduanjin exercise on quality of life among older adults.

This meta-analysis comprised four studies with 389 individuals (24, 27, 28, 39). Overall, Baduanjin exercise was associated with a significant improvement in quality of life among older adults compared with the control condition (*SMD* = 0.73, 95% *CI*: 0.29–1.18) (Figure 6). Detailed results of the subgroup analyses are provided in Supplementary Table 5. No statistically significant differences were identified across subgroups defined by health status, exercise frequency, measurement instruments, or age categories (*P* for subgroup differences > 0.05). The stability of the pooled estimates was further validated by sensitivity analysis, suggesting that the overall results were reliable (Supplementary Figure 7).

#### 3.4.4 Body composition

**Figure 7:**
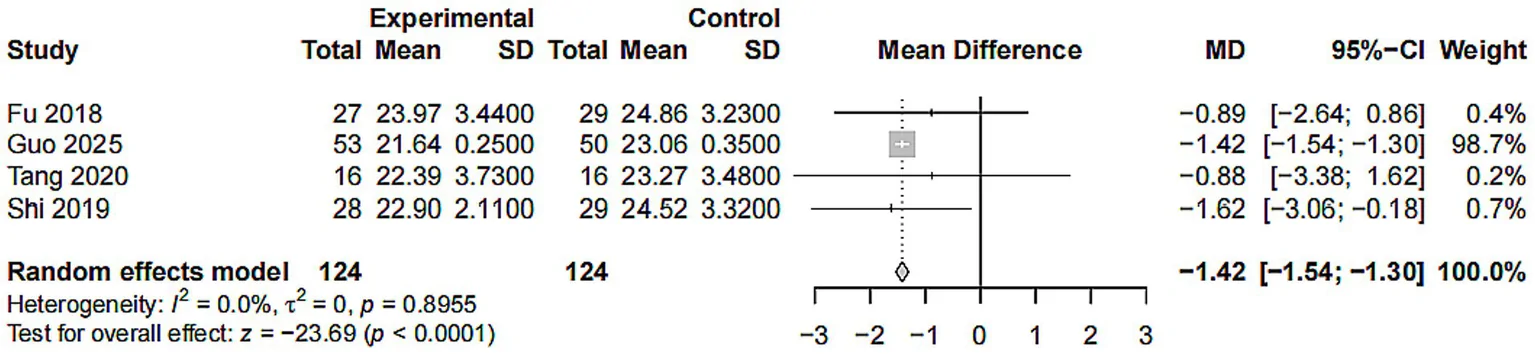
Meta-analysis of Baduanjin exercise on body mass index among older adults.

This meta-analysis comprised four studies with 248 individuals (26, 27, 30, 32). Overall, Baduanjin exercise was associated with a significant reduction in body mass index (BMI) among older adults compared with the control condition (*MD* = −1.42, 95% *CI*: −1.54 to −1.30) (Figure 7). Supplementary Table 6 presents specific results from the subgroup analysis. No statistically significant differences were detected across subgroups defined by health status, age categories, or session duration (*P* for subgroup differences > 0.05). Sensitivity analyses further confirmed the stability of the pooled estimates, indicating that the overall results were robust (Supplementary Figure 8).

The meta-analysis assessing waist circumference comprised two studies with 226 participants (26, 37). Overall, Baduanjin exercise was associated with a significant reduction in waist circumference among older adults compared with the control condition (*MD* = −3.52, 95% *CI*: −5.16 to −1.87) (Supplementary Figure 9). Likewise, two studies with a combined sample of 226 participants were analyzed for hip circumference (26, 37). The pooled estimates showed that Baduanjin exercise significantly decreased hip circumference relative to the control group (*MD* = −1.74, 95% *CI*: −1.94 to −1.54) (Supplementary Figure 10).

#### 3.4.5 Handgrip strength

**Figure 8:**
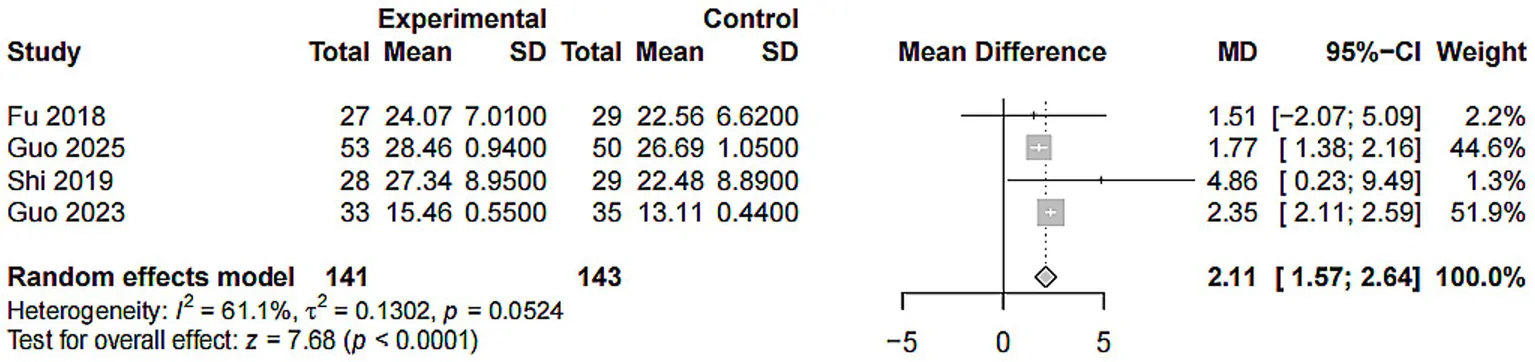
Meta-analysis of Baduanjin exercise on handgrip strength among older adults.

This meta-analysis comprised four studies with 141 individuals (26, 27, 30, 39). Overall, Baduanjin exercise was associated with a significant increase in handgrip strength among older adults compared with the control condition (*MD* = 2.11, 95% *CI*: 1.57–2.64) (Figure 8). Supplementary Table 7 presents specific results from the subgroup analysis. A greater improvement in handgrip strength was observed in participants aged ≥70 years than in those aged <70 years (*MD* = 2.35, 95% *CI*: 2.11–2.59 vs. *MD* = 1.79, 95% *CI*: 1.41–2.17). No statistically significant differences were detected across other subgroup categories, including health status and session duration (*P* for subgroup differences > 0.05). The stability of the pooled values was further validated by sensitivity analysis, suggesting that the general conclusions were solid (Supplementary Figure 11).

## 4 Discussion

This systematic review and meta-analysis indicates that Baduanjin exercise is associated with significant improvements across a range of health outcomes in older adults. Overall, the evidence suggests that Baduanjin contributes to reductions in PSQI scores, depressive symptom severity, BMI, waist circumference, and hip circumference, while simultaneously promoting better quality of life and greater handgrip strength. Although certain variables, such as assessment tools and age stratification, appeared to influence effect estimates in specific analyses, the majority of subgroup comparisons were not statistically significant. These findings suggest that Baduanjin may represent a promising mind–body exercise for promoting healthy aging among older adults. To our knowledge, this is one of the most comprehensive meta-analyses to simultaneously evaluate the effects of Baduanjin on sleep quality, depressive symptoms, quality of life, handgrip strength, and body composition among older adults.

Compared with previous reviews that primarily focused on single outcomes such as sleep quality or depressive symptoms, the present study provides a broader evaluation of both psychological and physical health outcomes in older adults. The findings of this study are generally consistent with earlier research examining how Baduanjin and other mind–body exercises affect psychological well-being and sleep quality. Previous studies have shown that Baduanjin and similar practices can significantly improve sleep, mental well-being, and physical functioning in older populations (15, 16). Moreover, relative to conventional exercise, mind–body interventions may produce stronger reductions in depressive symptoms, potentially due to their focus on relaxation and mindfulness-oriented regulation (40).

Nevertheless, some evidence has reported divergent findings. For example, certain studies indicate that conventional exercise may be more effective than mind–body approaches in reducing depressive symptoms among younger individuals (41). Such inconsistencies may be attributed to variations in exercise preferences and physiological characteristics across age groups. Older adults, often constrained by reduced physical capacity, are more likely to favor low-intensity, slow-paced activities, whereas younger individuals tend to prefer more vigorous and fast-paced forms of exercise (42). As a result, mind–body interventions that emphasize relaxation and attentional focus may yield greater psychological benefits in older adults, thereby enhancing overall quality of life. In addition, Baduanjin represents a practical and accessible form of physical activity that requires neither specialized equipment nor dedicated facilities, allowing it to be performed in both home and group settings (43). Compared with more physically demanding exercises, such as running or high-intensity aerobic training, which may be unsuitable for frail individuals, the gentle and low-impact characteristics of Baduanjin make it a safer option while still facilitating relaxation through controlled breathing (44). Taken together, these features further support the appropriateness of Baduanjin for older adults and underscore its potential as an effective strategy for promoting healthy aging.

The 2020 World Health Organization (WHO) guidelines on physical activity and sedentary behavior emphasize that engaging in regular physical activity yields substantial health benefits across individuals of all ages and functional abilities, including enhanced mental health, improved physical performance, and better overall quality of life (45). Consistent with these recommendations, the present study found that Baduanjin, a traditional mind–body exercise, was linked to notable improvements in a range of physical and psychological outcomes among older adults.

The underlying mechanisms through which Baduanjin exerts its effects are likely multifactorial, involving both physiological and psychological processes. To begin with, exercise may alleviate depressive symptoms by influencing neurotransmitter activity. Baduanjin practice may promote the release of neuroactive substances capable of crossing the blood–brain barrier and increase the expression of brain-derived neurotrophic factor (BDNF), thereby facilitating hippocampal neurogenesis and improving emotional regulation (46). In addition, Baduanjin integrates coordinated body movements, controlled breathing, and focused attention; such mind–body interaction may enhance parasympathetic tone while reducing sympathetic activation, contributing to relaxation and improved psychological well-being and quality of life (9). Furthermore, slow and controlled breathing accompanied by sustained attention may regulate autonomic nervous system function, relieve anxiety and psychological tension, and contribute to improved sleep quality (15).

Beyond autonomic regulation, circadian rhythm stabilization may represent another potential mechanism underlying the beneficial effects of Baduanjin on sleep-related outcomes. A randomized controlled trial conducted by Kung et al. reported that a 12-week Baduanjin intervention significantly increased interdaily stability of circadian rhythms, accompanied by improvements in sleep quality and traditional Chinese medicine body constitution. These findings suggest that Baduanjin may help synchronize biological rhythms, promote healthier sleep–wake patterns, and contribute to favorable changes in body constitution, thereby enhancing overall health status and sleep-related outcomes (47).

Considering the bidirectional association between sleep and emotional states, better sleep may, in turn, promote overall mental well-being (48). Additionally, from a physiological perspective, as a low- to moderate-intensity exercise modality, Baduanjin may positively influence body composition by stimulating lipid metabolism and limiting fat accumulation. Reductions in central and peripheral fat distribution as well as a lower body mass index (BMI) could result from these effects (49). Moreover, the repetitive upper-limb movements and hand coordination involved in Baduanjin practice may contribute to improvements in muscle strength, including handgrip strength, through enhanced neuromuscular activation and regular functional use of the upper extremities. Such improvements in muscle strength and physical function may be especially relevant to older adults, as lower handgrip strength has been consistently associated with frailty, disability, reduced functional independence, and adverse health outcomes in ageing populations (50).

Exploratory subgroup analyses suggested potential variations across different assessment instruments, with studies using the Geriatric Depression Scale (GDS) tending to report larger effect sizes than those employing the SDS or HADS. This finding may partly reflect the fact that the GDS was specifically designed for older populations and could be more sensitive to subtle emotional changes in this age group (51). In addition, participants aged ≥70 years appeared to show greater improvements in depressive symptoms and handgrip strength. This finding aligns with previous studies indicating that older adults may derive greater functional and psychological benefits from low-intensity mind–body exercise interventions, possibly due to their greater need for adaptable and low-impact forms of physical activity (52). However, these subgroup findings should be interpreted cautiously because several subgroup analyses were based on a limited number of studies. Future studies with larger sample sizes and more standardized assessment methods are needed to further clarify whether age and assessment tools influence the effectiveness of Baduanjin interventions.

Several limitations of this study should be considered. First, considerable heterogeneity existed across the included studies, which may be explained by differences in Baduanjin intervention characteristics (such as training frequency, session length, overall duration, and whether supervision was provided), as well as variability in participant health status and study design. Furthermore, the majority of the included research were carried out in China, which limits the global generalizability of the findings and presents a potential geographic bias. Given the deep cultural familiarity, historical roots, and widespread societal acceptance of Baduanjin within Chinese populations, participant compliance and intrinsic motivation may have been substantially higher than what would be observed in alternative cultural environments. Therefore, the generalizability of the findings to non-Chinese populations should be interpreted cautiously. Additionally, a number of studies did not include active control groups, with control participants typically receiving usual care or health education only. As a result, the potential influence of non-specific effects, including attention effects or social interaction, cannot be entirely ruled out. Moreover, the absence of long-term follow-up data in many studies limits the ability to assess the sustainability of the intervention effects and may affect the overall reliability of the findings. Variations in outcome measurement tools across studies may further reduce the comparability of results. Furthermore, the populations included in this analysis were heterogeneous with respect to health status, encompassing both generally healthy older adults and individuals with chronic conditions. Differences in baseline health status may influence the magnitude of intervention effects and limit the generalizability of the findings.

In order to establish the global generalizability of these outcomes, future research must expand beyond China through well-controlled randomized trials involving geographically and culturally diverse populations. Future large-scale, multicenter trials with rigorous methodological safeguards—specifically including robust allocation concealment and objective blinding of outcome assessors where double-blinding is structurally unfeasible—are urgently needed to strengthen the global evidence base. Standardized Baduanjin intervention protocols with explicitly operationalized parameters for exercise frequency, session duration, metabolic intensity, and qualified supervision strategies should be established to enhance cross-study comparability and facilitate accurate replication across international research centers.

Additionally, longer follow-up assessments are required to confirm whether the observed benefits for sleep quality, depression, and body composition persist over time. Subsequent studies should supplement subjective scales with objective, gold-standard measures—including actigraphy, polysomnography, DXA scans, and relevant biological markers. Combining these approaches will minimize reporting bias and provide deeper mechanistic insights into how Baduanjin optimizes health outcomes in aging population.

## 5 Conclusion

This systematic review and meta-analysis indicate that Baduanjin exercise significantly improves sleep quality, depressive symptoms, quality of life, body composition, and handgrip strength in older adults. As an accessible, low-intensity mind–body intervention, it represents a promising strategy for aging populations. However, due to high heterogeneity and methodological limitations across the included studies, these findings should be interpreted with caution. Large-scale, rigorous randomized controlled trials with diverse cohorts and longer follow-up periods are required to confirm these long-term benefits and establish Baduanjin’s comparative efficacy against other exercise modalities.

## Data Availability

The datasets analyzed in this study were derived from previously published articles, all of which are cited in the reference list of the manuscript.
