# Correction: Effects of Baduanjin exercise on sleep quality, depressive symptoms, quality of life, and body composition in older adults: a systematic review and meta-analysis of randomized controlled trials

**DOI:** 10.3389/fpubh.2026.1954762

**Published:** 2026-08-10

**Authors:** Frontiers Production Office

**Affiliations:** Frontiers Media SA, Lausanne, Switzerland

**Keywords:** Baduanjin, exercise, meta-analysis, older adults, systematic review

Reference 14 should have been reference 23, and vice versa. This error also affects where they should have been cited in the article text, throughout the Results section and in Table 1. The reference details and their in-text citations have been corrected.

Reference 14 is:

14. Dong J, Wang D, Li H, Ni H. Effects of different Chinese traditional exercises on sleep quality and mental health of adults: systematic review and meta-analysis. *Sleep Breath*. (2024) 28:29–39. doi: 10.1007/s11325-023-02881-6

Reference 23 is:

23. Chen MC, Liu HE, Huang HY, Chiou AF. The effect of a simple traditional exercise programme (Baduanjin exercise) on sleep quality of older adults: a randomized controlled trial. *Int J Nurs Stud*. (2012) 49:265–73. doi: 10.1016/j.ijnurstu.2011.09.009

The original version of this article has been updated.

