# Supplementary material for: Effects of Baduanjin exercise on sleep quality, depressive symptoms, quality of life, and body composition in older adults: a systematic review and meta-analysis of randomized controlled trials
Source: Front Public Health. 2026 Jul 30;14:1882039. doi: 10.3389/fpubh.2026.1882039 (PMC13501274; doi:10.3389/fpubh.2026.1882039)
Supplement: Supplementary file 1 [file Supplementary_file_1.DOCX]

**Supplementary Table 1. Search strategy for the seven databases (search updated to 3 April 2026)**

| Pubmed | | |
| --- | --- | --- |
| No. | Query | Results |
| 1 | ((((Baduanjin[Title/Abstract]) OR (Ba duan jin[Title/Abstract])) OR (eight brocade[Title/Abstract])) OR (eight section brocade[Title/Abstract])) OR (eight section brocades[Title/Abstract]) | 551 |
| 2 | (((((((((((((Aged[MeSH Terms]) ) OR (Aged, 80 and over[MeSH Terms])) ) OR (aged*[Title/Abstract])) OR (ageing*[Title/Abstract])) OR (aging*[Title/Abstract])) OR (old*[Title/Abstract])) OR (older adult*[Title/Abstract])) OR (geriatr*[Title/Abstract])) OR (gerontology*[Title/Abstract])) OR (elder*[Title/Abstract])) OR (senior*[Title/Abstract])) OR (older generation*[Title/Abstract]) | 5,975,994 |
| 3 | (randomized controlled trial [pt] OR controlled clinical trial [pt] OR randomized [tiab] OR placebo [tiab] OR clinical trials as topic [mesh:noexp] OR randomly [tiab] OR trial [ti]) NOT (animals [mh] NOT humans [mh]) | 1,648,733 |
| 4 | #1 AND #2 AND #3 | 166 |

| Cochrane library | | |
| --- | --- | --- |
| No. | Query | Results |
| 1 | (Baduanjin):ti,ab,kw OR (Ba duan jin):ti,ab,kw OR (eight brocade):ti,ab,kw OR (eight section brocade):ti,ab,kw OR (eight section brocades):ti,ab,kw | 632 |
| 2 | MeSH descriptor: [Aged] explode all trees | 729,827 |
| 3 | MeSH descriptor: [Aged, 80 and over] explode all trees | 92,915 |
| 4 | (aged OR aged, 80 and over OR aged people OR aged person OR aged subject OR elderly people OR elderly person OR elderly subject OR senior citizen OR ag*ing OR older OR ‘older adult?' OR older generation OR senior OR geriatr* OR geriatric medicine OR geriatric practice OR sociogeriatrics OR gerontolog* OR elder* OR old):ti,ab,kw | 850,728 |
| 5 | #2 OR #3 OR #4 | 854,827 |
| 6 | (randomized controlled trial OR randomized OR randomised OR randomization OR randomisation OR rct OR randomly OR placebo):ab,ti,kw | 1,561,725 |
| 7 | #1 AND #5 AND #6 | 268 |

| Embase | | |
| --- | --- | --- |
| No. | Query | Results |
| 1 | ('Baduanjin':ti,ab,kw  OR 'Ba duan jin':ti,ab,kw  OR 'eight brocade':ti,ab,kw  OR 'eight section brocade':ti,ab,kw  OR 'eight section brocades':ti,ab,kw) | 704 |
| 2 | ('aged'/exp OR 'aged':ti,ab OR 'aged people':ti,ab OR 'aged person':ti,ab OR 'aged subject':ti,ab OR 'elderly people':ti,ab OR 'elderly person':ti,ab OR 'elderly subject':ti,ab OR 'senior citizen':ti,ab OR 'older adults'/exp OR older) AND generation:ti,ab OR 'geriatrics'/exp OR 'geriatric aspects':ti,ab OR 'geriatric medicine':ti,ab OR 'geriatric practice':ti,ab OR 'geriatrics':ti,ab OR 'geriatry':ti,ab OR 'sociogeriatrics':ti,ab OR 'gerontology'/exp OR ag$ing:ti,ab OR older:ti,ab OR 'older adult$':ti,ab OR senior:ti,ab OR geriatr*:ti,ab OR gerontolog*:ti,ab OR elder*:ti,ab OR old:ti,ab | 3,844,349 |
| 3 | ('clinical trial'/de OR 'controlled clinical trial'/de OR 'randomized controlled trial'/de OR 'randomized controlled trial (topic)'/de) | 2,656,387 |
| 4 | #1 AND #2 AND #3 | 155 |

| Web of science | | |
| --- | --- | --- |
| No. | Query | Results |
| 1 | TS=(Baduanjin OR "Ba duan jin" OR "eight brocade" OR "eight section brocade" OR "eight section brocades") | 654 |
| 2 | TS=(Aged OR "Aged, 80 and over" OR aged* OR ageing* OR aging* OR old* OR "older adult*" OR geriatr* OR gerontology* OR elder* OR senior* OR "older generation*") | 6,537,727 |
| 3 | TS= clinical trial* OR TS=research design OR TS=comparative stud* OR TS=evaluation stud* OR TS=controlled trial* OR TS=follow-up stud* OR TS=prospective stud* OR TS=random* OR TS=placebo* OR TS=(single blind*) OR TS=(double blind*) | 8,232,494 |
| 4 | #1 AND #2 AND #3 | 222 |

| CINAHL | | |
| --- | --- | --- |
| No. | Query | Results |
| 1 | XB ("Baduanjin" OR "Ba duan jin" OR "eight brocade" OR "eight section brocade" OR "eight section brocades") | 258 |
| 2 | ((MH "Aged+") OR (MH "Aged, 80 and Over+") OR (MH “Gerontologic Nursing+” OR MH “Gerontologic Care”) OR MH (“Senior Centres”)) OR XB (aged OR ag#ing OR aged people OR aged person OR aged subject OR elderly people OR elderly person OR elderly subject OR older OR older adult* OR older generation OR senior OR geriatr* OR gerontolog* OR elder* OR old) | 1,446,768 |
| 4 | #1 AND #2 | 99 |

| CNKI | | |
| --- | --- | --- |
| No. | Query | Results |
| 1 | (TI='八段锦' or AB='八段锦' ) | 4,169 |
| 2 | (TI='老年' or AB='老年' ) | 1,268,160 |
| 3 | #1 AND #2 | 518 |

| Wanfang | | |
| --- | --- | --- |
| No. | Query | Results |
| 1 | (TI='八段锦' or AB='八段锦' ) | 4,617 |
| 2 | (TI='老年' or AB='老年' ) | 978,122 |
| 3 | #1 AND #2 | 714 |

# Supplementary Table 2. Subgroup analyses of the effects of Baduanjin on sleep quality

| Subgroup | Category | k | I² (%) | P for heterogeneity | MD (95% CI) | P for subgroup differences |
| --- | --- | --- | --- | --- | --- | --- |
| Health status | General population | 3 | 62.6 | 0.069 | -3.14 (-4.04, -2.23) | 0.254 |
|  | Sleep disorders | 3 | 53.3 | 0.118 | -2.11 (-3.06, -1.16) |  |
|  | Chronic disease | 5 | 70.2 | 0.009 | -3.04 (-4.11, -1.96) |  |
| Session duration | ≤30 min | 5 | 74.8 | 0.003 | -3.22 (-4.46, -1.98) | 0.554 |
|  | 31–45 min | 3 | 0.0 | 0.389 | -2.84 (-3.47, -2.21) |  |
|  | ≥60 min | 3 | 72.0 | 0.028 | -2.34 (-3.39, -1.29) |  |
| Age | <70 years | 4 | 71.9 | 0.014 | -3.14 (-4.32, -1.96) | 0.501 |
|  | ≥70 years | 7 | 62.1 | 0.015 | -2.66 (-3.39, -1.94) |  |

**Supplementary Table 3. Meta-regression analysis for sleep quality**

| Parameters | Coefficient (β) | SE | z value | p value | 95% CI |
| --- | --- | --- | --- | --- | --- |
| Intercept | −3.61 | 1.04 | −3.49 | 0.001 | −5.65, -1.58 |
| Intervention duration | 0.02 | 0.02 | 0.80 | 0.423 | −0.03, 0.07 |

Meta-regression analysis indicated that intervention duration was not significantly associated with the effect size (β = 0.020, 95% CI: −0.029 to 0.068, p = 0.423) and did not explain the between-study heterogeneity (R² = 0.00%).

# **Supplementary Table 4. Subgroup analyses of the effects of Baduanjin on depressive symptoms**

| Subgroup | Category | k | I² (%) | P for heterogeneity | MD (95% CI) | P for subgroup differences |
| --- | --- | --- | --- | --- | --- | --- |
| Health status | General population | 1 | – | – | -0.74(-1.11,-0.36) | 0.580 |
|  | Chronic disease | 3 | 77.2 | 0.013 | -0.94(-1.57,-0.32) |  |
| Measurement | HADS | 1 | – | – | -0.74(-1.11,-0.36) | 0.011 |
|  | SDS | 2 | 0.0 | 0.766 | -0.64(-0.97,-0.32) |  |
|  | GDS | 1 | – | – | -1.61(-2.16,-1.06) |  |
| Age | <70 years | 3 | 0.0 | 0.895 | -0.68 (-0.93, -0.44) | 0.003 |
|  | ≥70 years | 1 | – | – | -1.61(-2.16,-1.06) |  |

Abbreviations: HADS, Hospital Anxiety and Depression Scale; SDS, Self-Rating Depression Scale; GDS, Geriatric Depression Scale.

# **Supplementary Table 5. Subgroup analyses of the effects of Baduanjin on quality of life**

| Subgroup | Category | k | I² (%) | P for heterogeneity | MD (95% CI) | P for subgroup differences |
| --- | --- | --- | --- | --- | --- | --- |
| Health status | General population | 1 | – | – | 0.62 (0.22, 1.01) | 0.266 |
|  | Sleep disorders | 1 | – | – | 0.34 (−0.01, 0.70) |  |
|  | Chronic disease | 2 | 83.1 | 0.015 | 1.03 (0.20, 1.85) |  |
| Exercise frequency | 3–5 times/week | 3 | 83.0 | 0.003 | 0.78 (0.14, 1.42) | 0.689 |
|  | ≥6 times/week | 1 | – | – | 0.63 (0.21, 1.04) |  |
| Measurement | WHOQOL-BREF | 2 | 83.9 | 0.013 | 1.02 (0.19, 1.85) | 0.213 |
|  | SF-36 | 2 | 4.2 | 0.307 | 0.46 (0.19, 0.74) |  |
| Age | <70 years | 2 | 0.0 | 0.973 | 0.62 (0.34, 0.91) | 0.649 |
|  | ≥70 years | 2 | 91.4 | 0.001 | 0.89(−0.22,1.99) |  |

Abbreviations: WHOQOL-BREF, World Health Organization Quality of Life-BREF; SF-36, 36-Item Short Form Health Survey.

# **Supplementary Table 6. Subgroup analyses of the effects of Baduanjin on BMI**

| Subgroup | Category | k | I² (%) | P for heterogeneity | MD (95% CI) | P for subgroup differences |
| --- | --- | --- | --- | --- | --- | --- |
| Health status | General population | 3 | 0.0 | 0.768 | −1.42(−1.53,−1.30) | 0.782 |
|  | Chronic disease | 1 | – | – | −1.62(−3.06,−0.18) |  |
| Session duration | ≤30 min | 2 | 0.0 | 0.786 | −1.42(−1.54,−1.30) | 0.767 |
|  | 31–45 min | 1 | – | – | −0.89(−2.64,0.86) |  |
|  | ≥60 min | 1 | – | – | −0.88(−3.38,1.62) |  |
| Age | <70 years | 3 | 0.0 | 0.808 | -1.42(-1.54,-1.30) | 0.673 |
|  | ≥70 years | 1 | – | – | -0.88(-3.38,1.62) |  |

# **Supplementary Table 7. Subgroup analyses of the effects of Baduanjin on handgrip strength**

| Subgroup | Category | k | I² (%) | P for heterogeneity | MD (95% CI) | P for subgroup differences |
| --- | --- | --- | --- | --- | --- | --- |
| Health status | General population | 2 | 0.0 | 0.887 | 1.77 (1.38, 2.15) | 0.239 |
|  | Chronic disease | 2 | 11.1 | 0.289 | 2.50 (1.35, 3.64) |  |
| Session duration | ≤30 min | 3 | 73.6 | 0.023 | 2.12 (1.56, 2.68) | 0.741 |
|  | 31–45 min | 1 | – | – | 1.51(−2.07, 5.09) |  |
| Age | <70 years | 3 | 0.0 | 0.423 | 1.79 (1.41, 2.17) | 0.014 |
|  | ≥70 years | 1 | – | – | 2.35 (2.11, 2.59) |  |

**Supplementary Figure 1 Sensitivity analysis of sleep quality**

**Supplementary Figure 2. Trim-and-fill funnel plot of sleep quality**

**A**

**B**

**C**

**D**

**E**

**F**

**G**

**Supplementary Figure 3. Forest plots of Baduanjin exercise on the seven PSQI components in older adults. (A) Subjective sleep quality; (B) Sleep latency; (C) Sleep duration; (D) Sleep efficiency; (E) Sleep disturbances; (F) Sleep medication; (G) Daytime dysfunction.**

**Supplementary Figure 4. Subgroup analysis of the effects of Baduanjin exercise on depressive symptoms in older adults according to measurement scales(HADS: Hospital Anxiety and Depression Scale; SDS: Self-Rating Depression Scale; GDS: Geriatric Depression Scale)**

**Supplementary Figure 5. Subgroup analysis of the effects of Baduanjin exercise on depressive symptoms in older adults according to age groups**

**Supplementary Figure 6. Sensitivity analysis for depressive symptoms**

**Supplementary Figure 7. Sensitivity analysis for quality of life**

**Supplementary Figure 8. Sensitivity analysis for body mass index (BMI)**

**Supplementary Figure 9. Meta-Analysis of Baduanjin Exercise on Waist Circumference Among Older Adults**

**Supplementary Figure 10. Meta-Analysis of Baduanjin Exercise on Hip Circumference Among Older Adults**

**Supplementary Figure 11. Sensitivity analysis for grip strength**
